# Exploring patients’ perspectives: a mixed methods study on Outpatient Parenteral Antimicrobial Therapy (OPAT) experiences

**DOI:** 10.1186/s12913-024-11017-9

**Published:** 2024-04-29

**Authors:** Sophie Peter, Charlotte Oberröhrmann, Holger Pfaff, Clara Lehmann, Kirsten Schmidt-Hellerau, Vanessa Brandes, Charlotte Leisse, Christoph Heinrich Lindemann, Peter Ihle, Jutta Küpper-Nybelen, Anna Hagemeier, Nadine Scholten

**Affiliations:** 1 Chair of General Practice II and Patient-Centredness in Primary Care, Institute of General Practice and Primary Care, Faculty of Health, Witten/Herdecke University, Witten, Germany; 2 University of Cologne, Faculty of Medicine and University Hospital Cologne, Institute of Medical Sociology, Health Services Research and Rehabilitation Science, Chair of Health Services Research, Cologne, Germany; 3 University of Cologne, Faculty of Human Sciences and Faculty of Medicine and University Hospital Cologne, Institute of Medical Sociology, Health Services Research and Rehabilitation Science Cologne, Cologne, Germany; 4 Center for Health Services Research Cologne, Cologne, Germany; 5 Department I of Internal Medicine, Medical Faculty, University Hospital Cologne, University of Cologne, Cologne, Germany; 6 Center for Molecular Medicine Cologne (CMMC), University of Cologne, Cologne, Germany; 7 German Center for Infection Research (DZIF), Bonn-Cologne, Germany; 8 Department II of Internal Medicine and Center for Molecular Medicine Cologne (CMMC),, University of Cologne, Faculty of Medicine and University Hospital Cologne, Cologne, Germany; 9 PMV forschungsgruppe, Faculty of Medicine and University Hospital Cologne, Cologne, Germany; 10 Institute of Medical Statistics and Computational Biology (IMSB), Faculty of Medicine and University Hospital Cologne, University of Cologne, Cologne, Germany

**Keywords:** Patient Reported Outcome Measures, Patient satisfaction, Outpatient Parenteral Antimicrobial Therapy, OPAT, Intravenous therapy, Bacterial/viral infections

## Abstract

**Background:**

Outpatient Parenteral Antimicrobial Therapy (OPAT), an alternative to inpatient intravenous antibiotic therapy, has shown benefits in international studies such as increased patient satisfaction. Because OPAT has been used only sporadically in Germany so far, no structured results on patients’ experiences and concerns regarding OPAT have yet been available. This study therefore aims to explore the experiences of OPAT patients in a pilot region in Germany.

**Methods:**

This is an observational study in a German pilot region, including a survey of 58 patients on their experiences with OPAT, and in-depth interviews with 12 patients (explanatory-sequential mixed-methods design).

**Results:**

Patients reported that they were satisfied with OPAT. That a hospital discharge was possible and anti-infective therapy could be continued in the home environment was rated as being particularly positive. In the beginning, many patients in the interviews were unsure about being able to administer the antibiotic therapy at home on their own. However, healthcare providers (doctors and pharmacy service provider staff) were able to allay these concerns. Patients appreciated regular contact with care providers. There were suggestions for improvement, particularly concerning the organization of the weekly check-up appointments and the provision of information about OPAT.

**Conclusions:**

Patients were generally satisfied with OPAT. However, the treatment structures in Germany still need to be expanded to ensure comprehensive and high-quality OPAT care.

**Trial registration:**

NCT04002453, https://www.clinicaltrials.gov/, (registration date: 2019–06-21).

**Supplementary Information:**

The online version contains supplementary material available at 10.1186/s12913-024-11017-9.

## Background

When hospitalisation is required solely to administer intravenous antibiotic therapy to treat an infectious disease, Outpatient Parenteral Antimicrobial Therapy (OPAT) offers a means to entirely circumvent hospitalisation or at least significantly reduce its duration [1, 2]. OPAT involves the delivery of approved parenteral antibiotic therapy outside of an inpatient hospital setting. Typically, a secure vascular catheter is inserted, allowing for the administration of the anti-infective therapy via infusion. OPAT can be carried out in various settings, including a general practitioner’s office, a specialised outpatient facility, or even the patient's own home [2]. Patients may self-administer or receive assistance from an informal caregiver or professional caregiver [2, 3]. Typical indications for OPAT include skin and soft tissue infection, bone infection, and endocarditis, which are often associated with the need for prolonged intravenous antibiotic therapy [2, 4]. Offering comparable efficacy to inpatient care, OPAT presents numerous advantages, including a lower risk of nosocomial infections [5, 6]. At the same time, costs can be saved by preventing or shortening hospital stays [4, 7–9]. OPAT is therefore an efficacious and safe alternative to inpatient treatment [10, 11]. Research on OPAT consistently underscores the high levels of patient satisfaction attributable to its seamless integration into their daily routines [8, 12–16]. OPAT is a standard care practice in many countries [3, 17, 18]. However, in Germany, there is a lack of healthcare infrastructure, regulations, and recommendations to support OPAT [1]. As a result, OPAT is only offered by few specialised centres or practices [2, 19]. There has been little research about patients’ experiences with OPAT in Germany, despite the fundamental and active role of patients in this mode of treatment. In the spirit of patient-centred outcome measurement, this article aims to answer the question: How is OPAT experienced and accepted by patients in an urban pilot region of Germany (Cologne metropolitan region)? In a nationwide comparison, Cologne has an advantage in the developement of an OPAT structures: an infectiological network (consisting, for example, of hospitals and outpatient infectiological practices) dedicated to patient care, education and training and also research, e.g. for OPAT [1].

## Methods

A prospective observational study as part of the K-APAT study (“outpatient parenteral antibiotic treatment in the metropolitan region of Cologne”), which scientifically evaluated the implementation of OPAT in a German pilot region (the Cologne metropolitan region), was conducted. Clinical data is published separately [20]. A detailed description of the study design can be found in the study protocol [21]. The study has been approved by the Institutional Review Board of the University of Cologne, Germany (19–1284-1). The study is a multi-centre study consisting of 5 hospitals and 5 outpatient practices with a focus on infectiology. All study centres were allowed to include patients in the study [21]. Patients who were considered suitable for OPAT by the infectious disease service were also enrolled in the patient satisfaction survey study after giving informed consent to OPAT. Inclusion criteria were therefore admission as an OPAT patient; a minimum age of 18 years; and written informed consent to the survey study. As the survey documents could only be provided in German, sufficient knowledge of German was mandatory. Data collection was carried out using an explanatory mixed-methods design between November 2019 and September 2021 [22]. The explanatory mixed-methods design allows a broad yet thorough understanding of the patient perspective [22]. Patients were invited to answer up to three surveys delivered by mail about their OPAT experience at three timepoints: T0 (before starting OPAT, 48 items), T1 (two weeks after starting OPAT, 51 items) and T2 (one week after finishing OPAT, 42 items). Most of the items were likert scaled. All questionnaires included one open ended question (“If you have any comments, please note them here:”). After the patients were enrolled in the study, a written questionnaire was sent to their homes if they had given their consent. The data was collected pseudonymously in returning the questionnaire to the research institute, which was not involved in the treatment of the patients. The questionnaires were developed based on the current literature [2, 11, 13, 23–29] and critically reviewed by the study team consisting of social scientists, healthcare researchers, and clinicians with expertise in infectious diseases. The questionnaires used in the K-APAT study were developed specifically for this research. A German version is available in the project's final report [30]. Both self-developed questions and validated instruments were used in the questionnaires. The final report also contains information on validated and self-developed items [30]. The questionnaires were tested for comprehensibility and adapted within the framework of ten cognitive pretests with healthy adult persons of different age groups (19 to 70 years old). Topics of the questionnaires are shown in Table 1 (Tab. 1). See the appendix for the English version of the questionnaire items used for this publication (Additional file 1). Data were analysed descriptively using Stata 17 software.

**Table 1:** Topics of the survey

Topics	T0	T1	T2
Reasons for choosing Outpatient Parenteral Antimicrobial Therapy (OPAT)	x		
Therapy confidence	x	x	
Patient education in the study centres	x		
Health status & health-related quality of life	x	x	x
Experience with intravenous antibiotic therapy	x		
Personality traits	x		
Sociodemographic data	x		
Sick leave	x	x	x
Ability to work	x	x	x
Social support	x		
Discharge from hospital		x	
Clarification and training by the pharmaceutical service company		x	
Outpatient follow-ups^a^		x	x
Subjective treatment success		x	x
OPAT handling		x	
Negative aspects of OPAT^a^		x	x
Assessment of the pharmaceutical service company			x
Treatment errors			x
Restrictions in everyday life			x
Final evaluation of OPAT^a^			x

^a^Items used for this paper

After the quantitative data collection, in-depth interviews were conducted with selected patients who answered the questionnaires beforehand. These patients had indicated in the written declaration of consent for the study that they would be interested in taking part in in-depth interviews once the questionnaire survey had been completed. The patients were selected for the interviews by the researchers using the pseudonymised data set. The researchers were not involved in the patients' medical care and were only familiar with the patients’ T0, T1 and T2 questionnaires. The researchers wanted to map the greatest possible variability of the patients, especially with regard to their experiences and satisfaction with OPAT. Patients were also selected for a balance in gender, age, duration of OPAT treatment, and overall satisfaction with OPAT. All 12 interviews took place in person (mostly at patients' homes) or by telephone, due to contact restrictions during the COVID-19 pandemic.

**Fig. 1:**
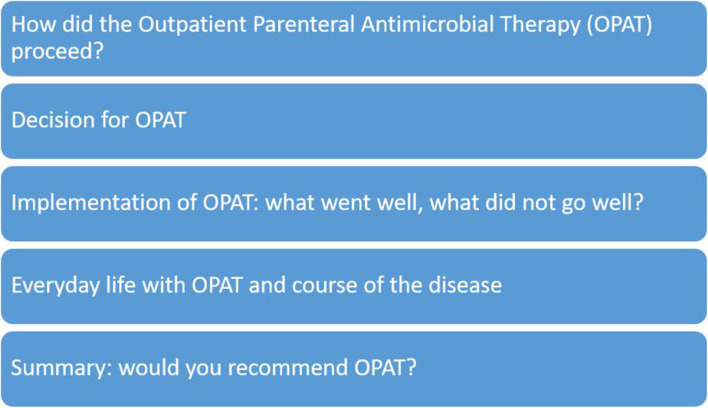
Topics of the interview guide

Topics of the interview guide are shown in Fig. 1 (Fig. 1). The English version of the interview guide is available as a Supplementary file (Additional file 2). Dimensions of the semi-structured interview guide were developed from the literature [11, 23–29] and expert feedback. The interview guide utilised in the K-APAT study was specifically developed for this research. The German version can be found in the final report [30].

The qualitative data were interpreted by means of a content analysis (inductive and deductive categories), which was carried out iteratively by SP and CO [31].

The participants received a financial incentive for completing the questionnaires and participating in the interviews.

## Results

Patients’ characteristics can be found in Table 2 (Tab. 2).

**Table 2:** Patients’ characteristics

	Questionnaires	Interviews
N	58^a^	12
Gender		
Male	44 (75.9%)	6 (50%)
Female	14 (24.1%)	6 (50%)
Non-binary	0	0
Age in years		
Mean ± SD	55.2 ± 15.6	59.8 ± 10.8
Range	21 – 93	40 – 73
Duration of OPAT in days Mean (min., max)	15 (min.: 5 max.: 127) [20]	27.3 (min.: 6; max.: 113)

^a^Screened patients: 94; patients treated with OPAT (per protocol): 77; patients excluded due to missing values in the questionnaire items used for this publication: 19

As stated in the publication on the projects’ clinical data, the most frequently treated infections were joint and bone infections (26% of the patients) and vertebral osteomyelitis (14% of the patients) [20]. All results (including the overall results of the three questionnaires and the interviews) and all items of the survey instruments are included in the final report of the K-APAT project [30].

### Results from the questionnaires

All 77 patients included in the study returned their questionnaires (response rate: 100%). 19 respondents did not answer at least one item of the questionnaire which are reported here. These patients were therefore excluded from the following analyses.

**Fig. 2:**
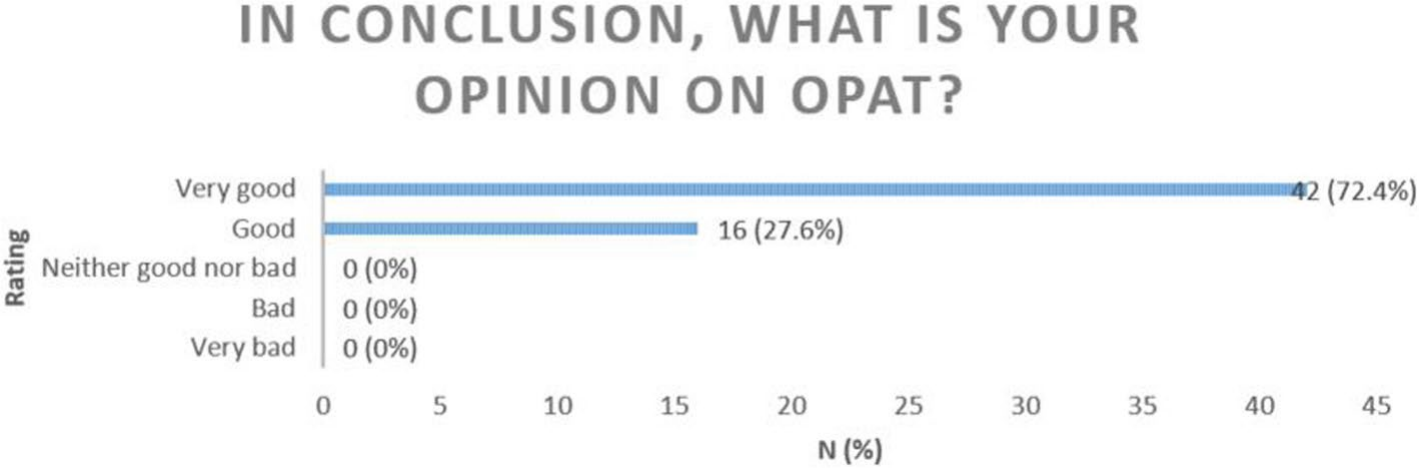
Opinions on OPAT

In the first and second questionnaire (T0 and T1) patients were asked about their experiences with OPAT subdivided in e.g. therapy confidence, experiences with intravenous antbiotic therapy and patient education (all topics are summarised in Table 1). In T2, all patients were asked to summarise their OPAT treatment. The aim was to obtain a final evaluation of their entire OPAT treatment. All respondents had a positive opinion on OPAT (Fig. 2).

**Fig. 3:**
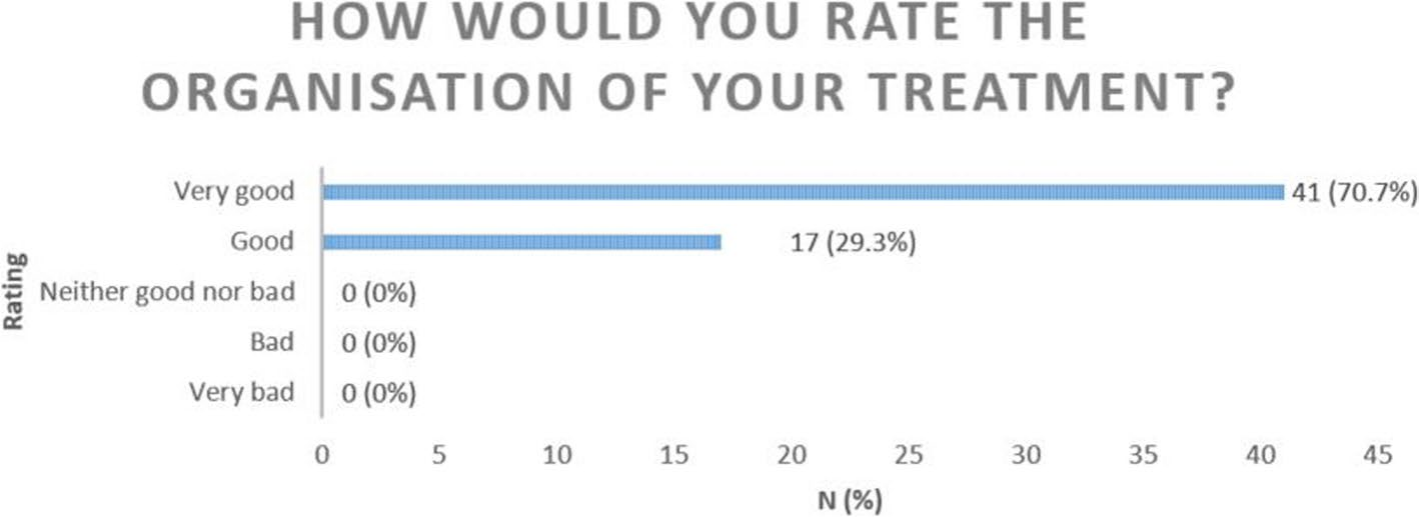
Rating of treatment organisation

Also as part of the final conclusion to their OPAT most patients rated the organisation of their treatment as good (29.3%) or very good (70.7%) (Fig. 3).

**Fig. 4:**
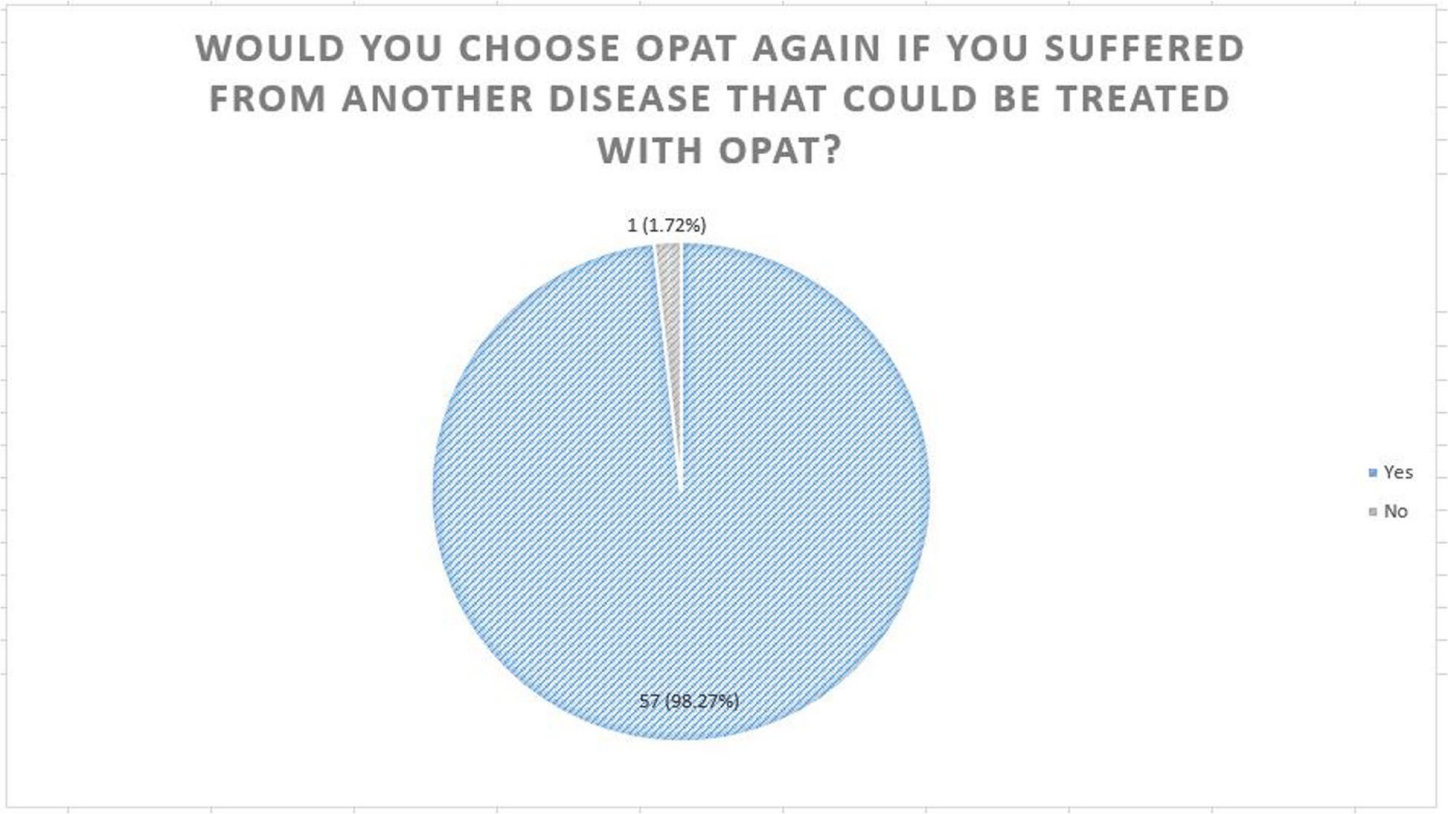
“Would you choose OPAT again if you suffered from another disease that could be treated with OPAT?”

**Fig. 5:**
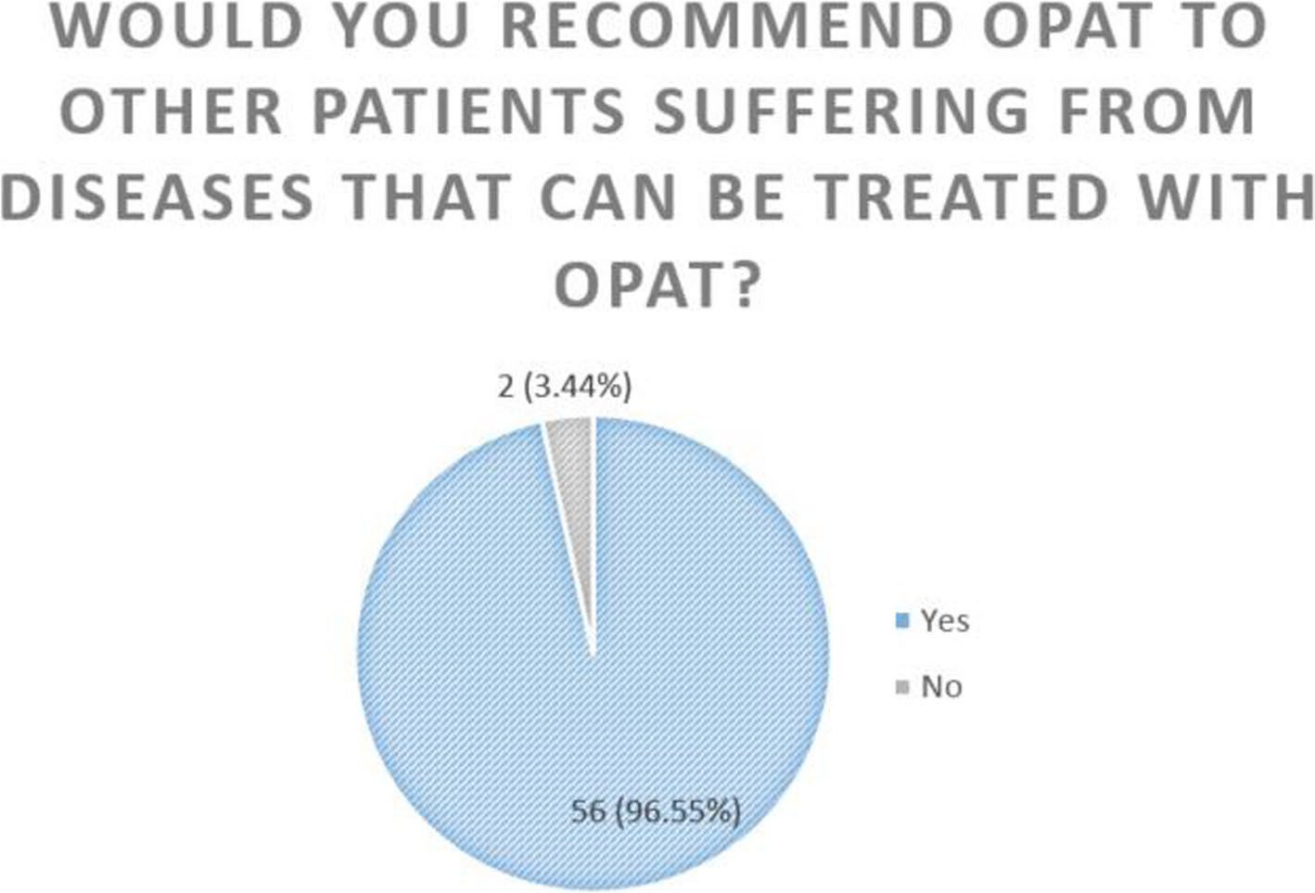
“Would you recommend OPAT to other patients suffering from diseases that can be treated with OPAT?”

The majority of patients (98.28%) would choose OPAT again if required. A similarly high proportion (96.55%) would recommend OPAT (Figs. 4 & 5).

Patients were asked to indicate their problems with OPAT using a Likert scale (strongly disagree, rather disagree, rather agree, strongly agree): Problems with material storage were infrequently reported, with only 10.34% strongly agreeing and 3.45% rather agreeing. A small number of individuals (1.72% strongly agree, 8.62% rather agree) reported feeling uncomfortable with the vascular catheter, while 13.9% tended to agree that the vascular catheter affected how they were perceived in public. Additionally, 12.07% strongly agreed and 18.97% rather agreed that they deliberately hid their vascular catheter in public. Eight people (3.45% strongly agree, 10.34% rather agree) reported complications during their treatment. None of the respondents reported any mistakes made by the medical staff, or that OPAT was an inappropriate treatment option for them (Table 3).

**Table 3:** Problems with OPAT

	Strongly agree N (%)	Rather agree N (%)	Rather disagree N(%)	Disagree N (%)
Problems with material storage	6 (10.34%)	2 (3.45%)	8 (13.79%)	42 (72.41%)
Feeling uncomfortable with the vascular catheter	1 (1.72%)	5 (8.62%)	10 (17.24%)	42 (72.41%)
The vascular catheter affects how I am perceived in public	0 (0%)	8 (13.9%)	10 (17.24%)	40 (68.97%)
I deliberately hide my vascular catheter in public	7 (12.07%)	11 (18.97%)	12 (20.69%)	28 (46.67%)
Complications occurred during OPAT treatment	2 (3.45%)	6 (10.34%)	6 (10.34%)	44 (75.86%)
The medical staff made mistakes in my OPAT treatment	0 (0%)	0 (0%)	5 (8.62%)	53 (91.38%)
OPAT was an inappropriate treatment option for me	0 (0%)	0 (0%)	4 (6.90%)	54 (93.10%)

### Results from the interviews

The results of the interviews are summarised in the Table 4 (Tab. 4).

**Table 4:** Interview results

Category	Sub-category	Results
Implementation of OPAT	Problems with OPAT	• most patients reported no problems, but some patients had problems with OPAT:
		• anxiety or uncertainty (e.g., fear of making mistakes when administering antibiotics; fear of venous catheter management because of the "at heart" location)
		• blockage of the catheter
		• being switched to oral medication
		• insecurities in handling
		• mechanic problems (e.g. taking the pump system off)
		• motor and cognitive limitations were suspected by interviewees as possible barriers to handling infusions
	Benefits of OPAT	• being discharged from the hospital early
		• OPAT offers a lot of freedom and independence
		• OPAT offers going back to “my normal life”
		• the treatment was predictable
		• OPAT is associated with cost savings for the healthcare system
		• venous catheters can stay in place for longer and does not need to be constantly replaced compared to peripheral catheters
Everyday life with OPAT and course of disease	Restrictions in everyday life due to OPAT	• minor restrictions in everyday life (e.g., sleeping on the side with the catheter is uncomfortable)
		• financial challenges (e.g. co-payment of the treatment)
		• suspected side effects (e.g., fatigue)
	Experiences with healthcare providers	• lack of awareness of OPAT among healthcare providers
		• organisational barriers (e.g. waiting times of check-ups)
		• venous catheter placement and removal took place in the hospital for all patients
		• OPAT briefing was good and adapted to the patients' needs
		• one patient reported she received too little information about OPAT from her physicians
		• OPAT information of healthcare providers were partially incomprehensible
		• support by the pharmacy service providers as well-organised
		• deliveries of the material were organised well
		• outpatient providers (e.g. General Practitioners) were only sporadically involved in OPAT provision

The interviews lasted between 20 and 58 min (mean length: 39 min).

Despite the overall positive evaluation of OPAT, some difficulties were identified during the interviews following explicit enquiries. Eight of the interviewees reported anxiety or uncertainty, especially at the beginning of their therapy (e.g., fear of making mistakes when administering antibiotics; fear of venous catheter management because of the “at heart” location). One patient commented: “This [OPAT] is excellent. Painless and great. However, it is a strange feeling considering the access is in the heart [sic]. (…) You do have a queasy feeling. (…) But it is great. I always thought: Well, what if it slips? But how is it supposed to slip? Slipping is not possible.” (patient 4). However, most fears quickly subsided after the start of therapy. One patient reported severe mechanical problems due to blockage of the catheter, which led to her being switched to oral medication for the last 3 days of her treatment: "Overall it went well and only few problems occurred. Sometimes it happened that, [patient paused and reformulated] So once I had difficulty taking it [the pump system] off again. We then had to use a pipe wrench.” (patient 8). However, most patients had no problems with the OPAT at all. One interviewee said: “And, yes, here at home I had no problems at all. Even the first application, I did it a bit more carefully, of course, just to not forget anything, to do everything right, and very quickly routine comes in.” (patient 5). Another patient summarised: “There were no problems of any kind. There was no pain. There were no incidents.” (patient 9). Motor and cognitive limitations were mentioned as possible barriers to handling infusions.

The interviewees reported numerous benefits of the OPAT. They could be discharged from the hospital early, which they associated with greater self-determination and higher quality of life than in the hospital. One patient stated: “So that [the OPAT] has already given me a lot of freedom.” (patient 11) and another patient mentioned: “As I said, I’m at home, I have my familiar surroundings, and then I feel like I’m back to a normal life and I have the disease under control. So it [the OPAT] was a good way to not have to keep going back to the doctor again and again.” (patient 6). All interviewees emphasised how important it was for their well-being to be at home. One patient explained: “That I can be at home. That’s important for me because I feel most comfortable at home. I have 100 TV channels, and at the hospital, I have five that I don’t really watch at home. Or I can eat whatever I feel like. Home is home.” (patient 7). Freedom and independence in a homecare setting were highly valued compared to the lack of freedom and dependence in inpatient care. A lot of patients explained that OPAT at home was easy to perform and helpful for recovery. In addition, the treatment was predictable: “That was ritualised. I then took a book at some point and read, sat down in the corner and read. I didn't move wildly, right?” (patient 3). Patients did not feel that OPAT interfered with their daily life. In the words of one interviewee:”So everything I do here at home, I’ve been able to do with the [venous catheter].” (patient 10). One patient added: “I would say top-notch. I can only recommend it to everyone. So who is fit enough: definitely. Yes. Although I was also in the hospital [patient paused and started a new sentence] They were all super nice, doctors, nurses, everyone. They really did their best, but home is home, right? And if you really have the opportunity, (…) immediately. So I would do that again immediately. Yes. I hope not that I need it again, but let’s just say I would do it in a heartbeat.” (patient 12). In addition, three patients who work in healthcare themselves stated that OPAT is associated with cost savings for the healthcare system. They emphasised that no hospitalisation or nursing service is required for this treatment. From their experience, the venous catheter can stay in place for longer and does not need to be constantly replaced compared to peripheral catheters, and they heal well.

The interviewees reported some restrictions in everyday life due to OPAT: e.g., sleeping on the side with the catheter is uncomfortable, financial challenges (e.g., co-payment for those with statutory insurance was not explained and cost coverage for those with private insurance was initially unclear, both should be communicated more clearly), insecurity in handling, and suspected side effects (e.g., fatigue, eczema, exhaustion that patients attributed to their OPAT) were also reported by the patients. Most patients did not describe any side effects: “And I didn’t have any side effects or anything. So not that I felt anything bad, that I felt bad, that I felt dizzy or anything. It went wonderfully.” (patient 1).

The interviewees also reported their experiences with healthcare providers. Often patients mentioned a lack of awareness of OPAT among healthcare providers in the hospital and organisational barriers such as waiting times. The venous catheter placement and removal took place in the hospital for all patients. Weekly check-ups were also primarily performed in a hospital outpatient clinic. One patient reported on her hospital stay as: “I already noticed that the doctor was a bit worried […] and then [he] told me to make sure that I observe the hygiene measures, that would be very important. I think there is a bit of fear when you hand things over to the patient: let's see what the patient does with it himself? Surely there are differences there, right?” (patient 8). The interviewed patients evaluated the support by the pharmacy service providers as well-organised. The deliveries of the material (e.g. bandages, material for cleaning catheters, disinfectant) were organised well and without problems. The study participants received a high quantity of material, often more than was required. The OPAT briefing was good and adapted to the patients' needs. Nevertheless, the interviewees reported that the information they got from their healthcare providers was partially incomprehensible (e.g. the step-by-step instruction of the OPAT is very complex with preparation of the material, cleaning of the skin and the catheter and connecting the pumps). One patient felt that she had generally received too little information about OPAT from her physicians. The patients liked the check-ups and the option to contact the healthcare providers by phone: “Yes, she [the pharmacy employee] also called me again herself, I think on the fourth day, which I thought was very nice, and asked if everything was okay, if I was getting along, if I needed anything else.” (patient 2). Outpatient providers such as General Practitioners and other outpatient care services played a minor role among respondents and were only sporadically involved in treatment. Overall, the patients rated the organisation of the OPAT as good.

## Discussion

### Main findings

In the K-APAT study, a prospective observational study, we aimed to investigate medical care with OPAT, focusing on the model region of Cologne. The goal was to assess the feasibility and success of implementing OPAT within the German healthcare system. The sub-study we presented here aimed to find out more about patient views on OPAT, their satisfaction, and their experience within the framework of a mixed-methods study.

In our study, OPAT had high levels of patient satisfaction, with nearly all patients expressing contentment. Other studies found comparable satisfaction with OPAT treatment with similar rates of recommendation and reporting that they would opt for OPAT again if necessary [13, 26]. Saillen et al. (2017) concluded that OPAT patients were “happy to take over some responsibility for their treatment” [26]. In general, patient satisfaction with OPAT is comparably high internationally [8, 12–16]. Quintens et al. (2020) also reported that all respondents were very satisfied with their OPAT treatment [32]. However, some patients in their study as well as in Berrevoets et al. (2018) also wanted more information about the OPAT and reported side effects attibuted to the OPAT [11, 32] as the patients in our interviews did too. Similarly as can be seen in our data, Saillen et al. (2017) reported that patients initially had concerns about the self-application of and reported on mechanical problems in handling the pump system [26].

Notably, the most satisfying aspect was that this form of treatment allowed patients to be discharged from the hospital. This effect does not only apply to the OPAT patients reported here: As hospitalisation is often perceived as a burden [33, 34], a lot of patients express satisfaction upon discharge from the hospital. The organisation of OPAT was largely perceived to be good. In particular, patients appreciated regular contact with healthcare providers (physicians and pharmacy service providers). It is important for the success of the therapy that patients feel comfortable and well cared for [11].

In countries where OPAT is well established, the number of patients participating in OPAT studies is significantly higher [35]. One Italian study showed that the COVID-19 pandemic supported the use of OPAT [10]. The number of participants in our study fell during the pandemic. We suspect that this is due to the heavy burden on hospital staff and outpatient practices.

### Strengths and limitations

It is important to consider limitations when interpreting the results of this study. It was conducted in the densely populated metropolitan region of Cologne: the findings may therefore not be fully representative of other regions in Germany (e.g. rural areas, regions without infectiological networks). But the region was well suited to conducting a feasibility study on OPAT due to the already established expertise of the regional, infectiological network. In addition, most of the study was conducted during the COVID-19 pandemic, which could have led to a number of limitations. For example, our participant number (*N* = 58) is low. Although all 77 patients we included in the study returned their questionnaires (response rate: 100%), due to missing values, we had to remove 19 patients from the dataset. We attempted to improve recruitment by having multiple study sites and conducting the study in a region that has a good OPAT care structure by national standards. Nevertheless, the case number limits the generalisability of the data. However, other international studies have reported similarly low participation rates [32, 36–38].

Compared to other studies our patient population was mostly younger (mean of survey patients: 55.2 years) except for the study presented by Al Shareef et al. (2022) with the same median age as in our study [38]: Chambers et al. (2019) reported on patients with a median age of 61 years [35]. Saillen et al. (2017), Staples et al. (2022) and Berrevoets et al. (2018) also reported a higher median age of 59 years, 62 years and even 68 years [11, 14, 26]. Treatment periods in our population (survey data: mean: 15 days, interview data: mean: 27 days) were higher than in other studies. Wolter et al. (2004) reported about 11 days, Hase et al. (2020) reported 13 days and Saillen et al. (2017) 8.5.days [8, 26, 36]. As is common in many other OPAT studies, our survey data also has a high proportion of male participants. [11, 14, 32, 36, 38].

It is particularly interesting that in the survey, OPAT was described as good or very good. More detailed insights from the interviews showed that, despite the overall good rating, patients had minor problems with OPAT. Thus, the mixed-methods design and the extensive interview data complement the quantitative data well, e.g. patients were able to report on their experiences in more detail and more individually than it was possible in the questionnaires. Thus, the mixed-methods design supported the breadth and depth of data [39] on patients' OPAT experiences.

We tried to include a wide range of patient experiences with OPAT in the in-depth interviews and have made efforts to achieve a gender and age balance of the interviewees. But as you can see in Table 2 the interviewees were a little older and had a longer treatment period of OPAT compared to the survey population. In addition, the proportion of men in the survey was higher than in the interviews. Despricption on the underlying diseases can be found in the publication on the clinical data [20]. Unfortunately, patients who would not recommend OPAT (*N* = 2) to others or tended to be less satisfied than average either did not consent to be contacted for an interview or were unwilling to be interviewed when asked to do so.

A professional proofreading service performed proofreading of this publication.

### Implications

In the long term, OPAT can be a suitable option for standard care.

OPAT not only increases patient satisfaction through cross-sector, indication-based and patient-oriented care, but also relieves the burden on hospitals by reducing inpatient bed days [1]. Moreover, the inpatient sector is facing mounting pressures arising from a scarcity of specialists, demographic shifts, and soaring costs. In response, healthcare policy is advocating for the expansion of the outpatient care model, guided by the principle of “outpatient care before inpatient care” [40].

The data has shown that the OPAT programme can be improved in the following ways to further increase patient satisfaction: Some patients reported they did not know about potential co-payments, which should be communicated more clearly. Waiting times for check-ups in hospitals or doctors' offices should also be reduced. Patients have expressed the wish for flyers or information material explaining the OPAT procedure as well as the handling of the intravenous access in more detail. This improvement has already been introduced into care at the study sites: Flyers and videos describing the OPAT procedure have been developed.

## Conclusions

This study shows a high level of patient satisfaction with OPAT. The preference for home-based treatment over hospital care is a key contributing factor. OPAT is still a relatively uncommon procedure in Germany, which is why there are still no nationwide structures for this treatment option. However, our studie shows that patients are satisfied with the care they received and that an expansion of the OPAT structures should therefore be considered. In the studied region, a quality infrastructure for OPAT exists, marked by specialists in specialised and interconnected outpatient clinics. Due to all of these advantages, it is important to further promote the use of OPAT and broaden these prerequisites by establishing adequate OPAT structures [41].

### Supplementary Information

**Supplementary Material 1.**

**Supplementary Material 2.**

## Data Availability

The datasets used and/or analysed during the current study are available from the corresponding author on reasonable request.

## Abbreviations

K-APAT: Outpatient parenteral antibiotic treatment in the metropolitan region of Cologne
OPAT: Outpatient Parenteral Antimicrobial Therapy
