# Supplementary material for: Exploring patients’ perspectives: a mixed methods study on Outpatient Parenteral Antimicrobial Therapy (OPAT) experiences
Source: BMC Health Serv Res. 2024 Apr 29;24:544. doi: 10.1186/s12913-024-11017-9 (PMC11057129; doi:10.1186/s12913-024-11017-9)
Supplement: Supplementary file 1 — Supplementary Material 1. [file 12913_2024_11017_MOESM1_ESM.pdf]

Additional file 1: Questionnaire items used for this publication

In conclusion, what is your opinion on OPAT?				
☐ Very bad	☐ Bad	☐ Neither good nor bad	☐ Good	☐ Very good

How would you rate the organisation of your treatment?				
☐ Very bad	☐ Bad	☐ Neither good nor bad	☐ Good	☐ Very good

Would you choose OPAT again if you suffered from another disease that could be treated with OPAT?	
☐ Yes	☐ No

Would you recommend OPAT to other patients suffering from diseases that can be treated with OPAT?	
☐ Yes	☐ No

I have problems storing the medication and dressings properly.			
☐ Strongly agree	☐ Rather agree	☐ Rather disagree	☐ Disagree

The venous access gives me an unpleasant feeling.			
☐ Strongly agree	☐ Rather agree	☐ Rather disagree	☐ Disagree

I have the feeling that the vascular catheter affects how I am perceived in public.			
☐ Strongly agree	☐ Rather agree	☐ Rather disagree	☐ Disagree

I deliberately hide my vascular catheter in public.			
☐ Strongly agree	☐ Rather agree	☐ Rather disagree	☐ Disagree

**There were complications during my treatment.**			
☐ Strongly agree	☐ Rather agree	☐ Rather disagree	☐ Disagree

**I had the impression that the medical staff made mistakes during my treatment.**			
☐ Strongly agree	☐ Rather agree	☐ Rather disagree	☐ Disagree

**I believe that outpatient parenteral antibiotic therapy was the wrong treatment option for me.**			
☐ Strongly agree	☐ Rather agree	☐ Rather disagree	☐ Disagree
