# Supplementary material for: Exploring patients’ perspectives: a mixed methods study on Outpatient Parenteral Antimicrobial Therapy (OPAT) experiences
Source: BMC Health Serv Res. 2024 Apr 29;24:544. doi: 10.1186/s12913-024-11017-9 (PMC11057129; doi:10.1186/s12913-024-11017-9)
Supplement: Supplementary file 2 — Supplementary Material 2. [file 12913_2024_11017_MOESM2_ESM.pdf]

Additional file 2: Interview guide

Introduction:

- Welcome, thank you for attending the interview
- Introduction of researchers
- K-APAT – project: abbreviation for "Outpatient parenteral antibiotic therapy (OPAT) in the Cologne metropolitan region", we collect data from a providers' & patients' perspective
- goal: researching the potential of OPAT & answering the question of whether OPAT can established as standard care
- "Rules" of the interview: Please describe your personal experiences, there is an audio recording, transcripts will be pseudomized
- We prepared different topics that will guide the conversation (Interview guide)
- We take notes during our conversation to possibly return to a topic
- Thematic focus: Your OPAT care
- Do you have questions before we start the interview?

Key question	Categories of the key question
**How did you experience your OPAT treatment?**	• How was your OPAT treatment? How did it go?<br>• We saw in your questionnaires… (reference to PROMs based on the questionnaires T0, T1, T2)
**Decision in favour of OPAT**	• Why did you choose OPAT and not inpatient care?<br>• Medical advice: Which information about OPAT did you get from the medical staff? Was OPAT recommended to you by your health care providers?<br>• Expectations of OPAT before treatment: What did you expect from the treatment? Did your expectations change during the course of treatment? Which advantages and disadvantages did you expect due to OPAT?<br>• Social support at home & environmental factors (contextual factors): Did you have support from your family/neighbours/other persons during the treatment?

**OPAT treatment: What went well, what didn't?**	• Involvement in the decision-making process / OPAT care: (How) were you involved in treatment decisions?<br>• Access to care / organisational hurdles: How did you experience the “first steps” in your OPAT treatment?<br>• Transition between inpatient and outpatient care: How was the hospital discharge?<br>• Medical care: How was the medical care?<br>• Medical resources/equipment: Did you get enough OPAT materials (e.g. dressing material, disinfectant, rinsing solution …)?<br>• Communication between health care providers (with each other, with the patient): How did you experience the communication of your health care providers? Did you have the feeling they discussed your treatment? How did they communicate with you?
**Daily life with OPAT / course of the disease**	• Everyday life: How was your everyday life with OPAT?<br>• Professional life: Did you work during your treatment?<br>• Handling the venous access: How did you manage the handling of the venous access?<br>• Course of the disease: How did your illness develop during the OPAT?<br>• Emergencies & Complications: Did you experience any emergency situations or complications?<br>• Well-being & quality of life: How did you feel during the treatment? How was your quality of life?<br>• Role as a patient: Did you feel involved in treatment decisions or were the decisions dictated by doctors/other health care providers?
**Summary: Would you recommend OPAT?**	• Based on your current state of knowledge: Would you choose OPAT again if needed? Would you recommend OPAT?
